# Introduction to mechanisms of neural circuit formation

**DOI:** 10.3389/fnmol.2013.00012

**Published:** 2013-05-13

**Authors:** Joshua A. Weiner, James D. Jontes, Robert W. Burgess

**Affiliations:** ^1^Department of Biology, University of IowaIowa City, IA, USA; ^2^Department of Neuroscience, The Ohio State UniversityColumbus, OH, USA; ^3^The Jackson LaboratoryBar Harbor, ME, USA

Much progress has been made in recent years toward understanding the underlying causes of neurodevelopmental disorders. Whereas catastrophic failures in early events such as cell fate specification or cell migration give rise to profound developmental defects including microcephaly and lissencephaly, more subtle conditions such as autism, intellectual disability or neuropsychiatric disorders increasingly appear to result from comparatively minor changes in neural circuit formation and function. The formation of proper neuronal circuitry relies on later developmental processes such as axon guidance, the arborization both of axons and their target dendrites, the recognition of appropriate synaptic partners, the establishment and maturation of synaptic connections, and the subsequent elimination of improper connections. The research topic presented here, “Mechanisms of Neural Circuit Formation,” addresses recent advances in our understanding of the cellular and molecular bases of these processes.

The papers in this volume generally fall into three broad areas of developmental neurobiology, including new techniques for the study of these processes: (1) cell adhesion molecules (CAMs) and their downstream roles in cell identity, recognition, and synaptic specificity; (2) axon guidance, formation of terminals, and dendritic arborization; and (3) formation of synaptic structures themselves, the essential final step in circuit formation which, nevertheless, remains subject to remodeling and plasticity throughout development and even in adult animals.

The volume opens with a review by Sokolowski and Corbin (2012), highlighting the importance of neuronal circuit formation for behavior through an examination of the development of the limbic circuitry. This is followed by a series of papers on CAMs: a review from Weiner and Jontes (2013), and a hypothesis paper from Yagi (2012) that describe the roles of protocadherins. These are followed by the new results of Prasad and Weiner (2011) on a requirement for γ-protocadherins in Ia afferent terminal formation in the spinal cord. Next is a review from Garrett et al. (2012) on the immunoglobulin (Ig) superfamily proteins Down Syndrome Cell Adhesion Molecule and related proteins (DSCAMs), addressing issues of molecular diversity, cell identity and cell adhesion as they contribute to synapse and circuit formation. Finally, Enriquez-Barreto et al. (2012) present original research on the function of the Ig superfamily member Neural Cell Adhesion Molecule (NCAM) in axon pathfinding and organization in the thalamocortical system.

This last paper transitions into a section focused on axon guidance, including both signaling and cytoskeletal mechanisms. Sakai and Kaprielian (2012) review the literature on the guidance of longitudinally projecting axons, a prominent focus of study in both vertebrates and invertebrates. An original paper by Leslie et al. (2012), examines the requirement of RhoA in sensory axon guidance. The relationship of axon guidance mechanisms to other processes involved in synaptic specificity is highlighted in a review of semaphorin signaling by Yoshida (2012). Finally, the importance of FGF22 in axon termination and synaptogenesis in the lateral geniculate is demonstrated by Singh et al.'s original research (2012).

The penultimate group of papers concerns mechanisms regulating the formation of pre-and post-synaptic structures. The assembly and plasticity of the presynaptic active zone is reviewed in Clarke et al. (2012). A review from Winnubst and Lohmann (2012) discusses the mechanisms and implications for synaptic clustering on target neurons. An original research paper from Murphy et al. (2012) investigates the developmental regulation of AMPA receptor trafficking proteins.

The E-book closes with two papers describing powerful new techniques for studying neural circuit formation that are revealing novel biology both in Drosophila, as described by Nose (2012), and in mice, as described in Yamagata and Sanes (2012).

Together, these papers provide a broad reflection of the state of our knowledge concerning the molecular cues that direct axon and dendrite development, promote the formation of synaptic connections, and allow the refinement of connectivity and plasticity during development. An interesting observation that comes from these studies is that few, if any, of the key molecules are single-purpose; each pathway is used at distinct stages of neural development and serves multiple functions. The extent to which these functions relate varies. For example, semaphorins broadly influence both axonal and dendritic development, perhaps not surprisingly given the large number of different ligands and receptors involved. Fibroblast Growth Factor family members (FGFs), critical players in a wide variety of developmental events in the early embryo, are much later re-purposed to regulate the formation of synaptic circuitry. CAMs like the γ-protocadherins and DSCAMs, regulate several key processes, including neuronal survival, dendrite arborization, and axonal targeting; here, it remains unclear whether these functions are achieved through common signaling pathways, or whether they represent distinct roles for these CAMs. Thus, despite the daunting complexity of the task, the cast of key players is not increasing as rapidly as the number of roles assigned to each cast member. This could facilitate future progress by focusing efforts on a smaller number of molecules. Conversely, efforts could be complicated by the need to dissect primary and secondary effects for each pathway. This highlights the need to be particularly rigorous in defining where, when and how the functions of these proteins are analyzed. As the methods and specificity of these analyses improve, so will our understanding of neural circuit formation.

Considering the finely-tuned complexity of the mature nervous system, the robustness of neurodevelopment is amazing; despite genetic and environmental variability during development, and the stochastic variation inherent in biological systems, most brains end up working pretty well. The experimental investigation of small circuits and defined aspects of neural circuit formation is facilitating progress in this area, as the papers in this volume demonstrate. However, a key challenge for the future is to integrate the information we are gaining on individual molecular pathways into a larger model in order to understand how the neural circuitry of the mature nervous system is assembled during development.
